# Science, technology, and innovation policy timing and nanotechnology entrepreneurship and innovation

**DOI:** 10.1371/journal.pone.0264856

**Published:** 2022-03-03

**Authors:** Jennifer L. Woolley, Nydia MacGregor

**Affiliations:** Department of Management and Entrepreneurship, Leavey School of Business, Santa Clara University, Santa Clara, California, United States of America; Gonbad Kavous University, ISLAMIC REPUBLIC OF IRAN

## Abstract

The timing of science, technology, and innovation (STI) policy initiatives is critical to the outcomes that they produce. This study examines the advantages and disadvantages of enacting STI policy investments early in a nascent domain of activity. Building on work across multiple disciplines, we propose a framework to better understand the temporal dynamics of STI policy. An examination of data on nanotechnology STI policy around the world shows that the timing and funding size is related to entrepreneurship and innovation in different ways. The findings reveal that countries that started funding national STI programs in nanotechnology later had a lower proportion of the total nanotechnology firms, patents, and publications in the world, which suggests some first-mover advantages to STI policy. However, this is only part of the story. Countries that had large programs after the technology had gained legitimacy had the opposite situation such that there was a higher proportion of the total nanotechnology firms, patents, and publication in the world and more nanotechnology-related patents per capita and firms relative to other firms in the country explicating some of the complexity of policy timing. We discuss how temporal considerations influence both the theory and practice of building systems of innovation.

## Introduction

Innovation and entrepreneurship have emerged as policy imperatives in many countries, backed by large budgets and considerable public attention. Policy makers in nations, states, regions, and cities have implemented a variety of initiatives with the hopes of bolstering existing regional innovation systems or shepherding new ones to improve economic growth and prosperity [1, 2]. Science, technology, and innovation (STI) policies are one set of government initiatives designed to support basic research, innovation, and commercialization of inventions [3–5]. STI policy can increase the amount of knowledge spillover [6], the speed and level of product and industrial emergence [7–9] and an area’s economic growth [10].

Such policies face challenges due to the long time horizons required for their effects to take hold [11] and the complex sets of interacting actors that are endemic to innovation and economic systems [12–14]. However, in nascent technological domains, with heightened uncertainty, the challenge is even greater. In early stages of technological evolution, policy makers must also decide *when* to enact STI policies [15]. If a country moves early, enacting initiatives while a scientific discovery or technology is still in flux, the funding to support such activities may foster discovery, speed the resolution of technological uncertainty and enhance the legitimacy of new industries and businesses in its economy. At the same time, acting too early is risky in that funding may be squandered during the tumultuous formative years of a technology’s development, which sometimes does not come to fruition. Creating policies too late may position the country to have missed opportunities. Thus, for countries, regions or cities that are attempting to build entrepreneurial ecosystems in emerging domains, the timing of STI policy implementation is crucial. This study examines the relationship between the timing of a country’s enactment of STI policies associated with a nascent technology domain, and the long-term entrepreneurship and innovation related to that technology that occurs. However, policy timing is “poorly recognized theoretical idea/concept in public policy shaping” ([15], p. 1).

Recent work on STI policies has focused on policy mix [16–19] and the ability to respond to grand challenges and sustainability [20]. Indeed, STI policies increase innovation and economic progress [21, 22] and assist in cultivating new markets and managing uncertainty (e.g., [23, 24]). Some work has looked at the accumulation of policies [25–27]; however, the question of policy timing remains largely neglected [15, 28]. Indeed, it is not clear if the countries that get involved in supporting the development of a nascent technology or innovation are the main benefactors or if other lagging countries can also benefit, if not catch up.

We examine the influence of STI policy on related entrepreneurial and innovation outcomes by applying the insights for firms from the first-mover perspective from strategic management to STI policy timing. Considering that firms are socially constructed institutions, extending the first-mover perspective to other social constructions, such as institutions, social movements, and policy, provides insight into the advantages and disadvantages that the timing of policy enactment may generate. We find several parallels between firms and countries that may shed light on how timing may influence long term outcomes. The study then applies these insights to almost 40 years of nanotechnology STI policies (1981–2017) throughout the world. Using a unique database of nanotechnology policies, activities, entrepreneurship, patents, and publications, we are able to examine how the early implementation of STI policy may benefit an emerging domain by fostering the development of an innovation system looking at several indicators of innovation and entrepreneurship: nanotechnology patenting, publication rates, and the presence of nanotechnology firms. We also explore potential disadvantages. By controlling for country specific economic and demographic conditions, we take into account many of the contextual influences. Results show that the timing of STI policy is significant and moving quickly may not always be the best bet. Indeed, countries that started later or built STI initiatives over time benefited in terms of venturing and innovation activities compared to countries with large and early programs.

This study has several theoretical contributions and policy implications. Firstly, this research provides a provocative lens by which to consider the timing of STI policy. Work examining STI policy spans several disciplines including economics, political science, business and management, sociology, and psychology—or what Morlacchi and Martin ([22], p. 572) call ‘a somewhat heterogeneous set of activities undertaken by a community of diverse actors.’ Appropriately, the first-mover perspective crosses several disciplines, and creates a common ground for improving the understanding of STI policy. Notably, we do not offer an exhaustive discussion of the connections between the first-mover perspective as applied to STI policy and all other relevant disciplines. Rather, in extending application of the first-mover perspective, we unpack some of the temporal dynamics of national systems of innovation and STI policy and offer compelling evidence to improve understanding in this vital policy area. We also consider the role of policy in the development of science and innovation in both developed and developing countries, an area that is relatively neglected [29]. By improving our knowledge of the temporal dimensions of STI policy, participants can better manage the inherent advantages and disadvantages therein to improve system development, technological advancement, and economic prosperity. This is particularly important for effectively attending to contextual constraints [12]. Finally, firms active in emerging technological domains can improve decision-making based on this framework.

The objective of this study is to explore the implications of applying the first-mover perspective to a country level of analysis. As such, the theoretical background section starts with a discussion literature and research on STI policy and the first-mover perspective as it is traditionally applied. Next, we extend the first-mover perspective to countries and investigate its implications for enacting STI initiatives while a technology is still being developed. The following section, Methods, describes the setting for the empirical study: nanotechnology in 71 countries. After addressing the findings, we consider the practical and theoretical implications, as well as limitations, of this approach.

## Theoretical background

Innovation and entrepreneurship have become policy imperatives throughout the world as they are considered critical to boosting productivity, economic health, and rejuvenation, which influence the competitiveness of cities, countries and regions [30]. Science, technology, and innovation (STI) policies are government initiatives designed to support basic research, technology development, and innovation commercialization and adoption [3–5, 17]. Policy makers design and enact STI policies to strengthen national (or regional) innovation systems and spur economic development [31, 32]. Conceptually, scholars have acknowledged several temporal characteristics relevant to the interplay between STI policies and innovative and economic activity [15]. Specifically, STI policies stem from a path dependent political process, with long and weak feedback loops [26, 33]. As in other policy arenas, STI policies emerge from a political process where each new generation of politicians inherits the decisions of the previous administration [34], building on the foundations laid by their progenitors [35]. Often, the success of a policy or program depends on the system and policies existing when it is implemented [36].

STI policies should, arguably, be enacted when a jurisdiction believes that the infrastructure necessary to support those policies exists. Moreover, the evolving nature of innovation systems means that the efficacy of various STI policy instruments also changes over time [26]. Successful STI policies build infrastructures that require long time horizons or development of strong market relationships. This suggests that countries or regions that enact innovation policies during the formation of a new technology or innovation earlier than other countries or regions, may be more likely to build a successful system of innovation in a nascent domain [27, 36]. While this pattern is important globally, emerging economies are moving faster with the objective becoming technology leaders instead of followers [37].

In spite of the generally acknowledged role of temporal dynamics, we know little empirically about how the timing of policy influences long-term outcomes. Woolley and Rottner [5] find that in the U.S., states with the first STI policies for nanotechnology also had higher rates of nanotechnology-related entrepreneurship. Similarly, Beaudry and Allaoui [38] estimated a time-related model of the influence of public financing for academic research in nanotechnology and find an exponential relationship over time between this type of STI funding and scientific productivity. An emerging stream of research has begun to examine the evolution of policy mixes over time, which considers experimentation and reaction by governments as institutions, industries, and technologies change [39, 40]. These limited studies suggest that temporal dimensions do affect the outcomes of STI policy initiatives, but the topic remains under-examined, particularly across countries. Specifically, it is unclear how the timing of STI policy influences the growth of an innovation system or advancements in science, technology, and innovation. In this regard, there is a question as to when a country becomes involved in supporting a nascent domain. For example, is it only the governments that support emerging scientific domains early that reap the benefits of related innovations, technologies, and firms or can later entrants also benefit?

Looking to other perspectives may offer insight into this line of inquiry. The first-mover perspective, which examines the timing of firm entry into a market, may offer such guidance. Applying a firm-level construct to a larger (innovation) system is not without precedent. Examples include industrial clusters [41] and regional advantages [42, 43], where the firm-level concept of *competitive advantage* describes analogous attributes of regional or national economic development. Governments are competitive entities that work to establish comparative advantages for their constituents, which can enable economic prosperity. Firms and government institutions are socially constructed bodies that both garner resources and create value to survive and remain relevant. Moreover, explicit in many policy makers’ efforts to implement STI policy is ‘the desire to move from the role of science and technology follower to first-mover’ ([37], p. 732). The following section explores the application of the first-mover advantage perspective to countries and regions.

### The first-mover advantage perspective: Firms and countries

First-mover firms are those that enter a market earlier than other firms—they are pioneers in the field. Lieberman and Montgomery [44] proposed that these pioneers may earn higher economic profits than firms that enter later due to advantages generated through technological leadership, scarce asset preemption, and heightened buyer switching costs. These mechanisms, along with a dose of luck and firm proficiency, can enable the firm to generate profits beyond that of later entrants [44]. Suarez and Lanzolla [45] provide an in-depth analysis of empirical work on the first mover advantage, and Nehrt [46] summarizes work on the maintenance of these advantages. Analogous to first-mover firms, first-mover countries are pioneering nations that are motivated to implement policies ahead of others to stimulate progress in a nascent science, technology, and innovation domain. Being able to enact a first-mover STI policy signals that the focal country is willing and able to take a chance and support an emerging domain of activity. Similarly, countries may enact STI policies early in a technology’s development if they already have the capacity within their borders (perhaps in an adjacent technology or domain) or because they have the desire to lead in the new domain when they have not previously. Both first-mover firms and countries operate in light of their historical legacies, existing inertia, and policies. Decision makers at both firms and governments must weigh the risks associated with moving early, while a technology is emerging, and that of not moving fast enough and failing to support infrastructure that enables new or continued economic growth. Table 1 summarizes the key characteristics of first-mover and later-stage STI policies, including risk, motivations and advantages and disadvantages. The similarities between first-mover firms and countries make further application of the perspective provocative.

**Table 1:** Key characteristics of first-mover and later-stage countries in nascent domains.

	First-Movers	Later-Stage
**Motivations**	Establish technological leadership and build nascent markets and industries	Build location-relevant technology and innovation to contribute to economy
**Characteristics**	Cutting-edge innovation initiatives	Follower initiatives
	Tend to be broad based	More focused scope
	Supports era of technological ferment	Support era of incremental change
	Require established basic infrastructure	Based on first-mover initiatives
**Advantages**	At the forefront of technology	Build on the shoulders of giants
	Build specific infrastructure for future innovation development	Lower R&D costs for basic science
	Can guide determination of dominant design	Standards and norms are established
	Involved in innovation diffusion	Technology established
	Scarce assets are preempted	Can use others’ templates
	Early beneficiaries become embedded in local innovation system	Benefit from policy and procedure lessons from previous countries’ successes and failures
	Can direct future innovation policy and standards assets	Lack of history means less inertia- more agile
	Early returns are basis for re-investment	Learn from failed initiatives
	Attract complementary	
	Builds and shapes reputation	
**Disadvantages & Risks**	Initiatives educate competitors	Lack history of activity and infrastructure in domain
	Resources used to build infrastructure	Relies on others work and foundation
	Build infrastructure that others use	Rely on standards established elsewhere that may not be optimum
	Technology uncertainty and risk resolution	Cannot change standards and norms
	Higher R&D costs	Option may prove too costly to switch
	Build standard practices and norms	May be too late to attract complementary assets
	Enables new entrants	Lack of reinvestment
	Provides template for later-stage	Lack of reputation in field
	Incumbent inertia makes new programs difficult to implement	
	Expend resources to overcome the lack of legitimacy	

#### First-mover advantages

Traditional first-mover advantages include technological leadership, scarce asset preemption, and heightened buyer switching costs. The advantage of technological leadership stems from learning the market early and attaining patents or trade secrets before other firms do. These mechanisms facilitate higher product adoption rates and erect barriers to market entry. In terms of countries, technological leadership advantages stem from enacting policies related to a nascent domain. Early policies support basic scientific research when no one knows the specific avenue of research that will succeed in the long-term. First-mover STI policies, therefore, often support a broad base of research and innovation [47] and can influence the direction of a technology’s development and be involved in establishing the norms, dominant designs and standards of the field [48]. As infrastructure building is a costly and time intensive endeavor, an early start can put the country at the forefront.

Learning and patenting activities enabled by early STI policies can create a forerunner stronghold of tacit knowledge and intellectual property (IP) in a country. This may, in turn, enable those in the country to influence the innovation’s design and increase the likelihood of technological leadership. Thus, first-mover STI policy provides resources that may lead to technological leadership within the policy’s domain of influence. For example, Japan’s central government integrated nanotechnology into their national Science and Technology Basic Plan in 1995, which guided their STI policy for the next four years. This lead helped the country establish technological dominance in the field and a prominent role in the subsequent international road mapping for nanotechnology development. Similarly, Foladori and Invernizzi [49] found that Argentina, Brazil, and Mexico benefited from starting nanotechnology initiatives earlier than other countries in Latin American by being able to build technology-specific capabilities that led to their technological leadership in the region.

First-mover firms can benefit from scarce asset preemption when they are able to gather and control existing scarce resources such as critical supplies, plants, and equipment [45] and specialized complementary assets [50]. Similarly, countries with early STI policies may enable and support the creation and lock-in of critical scarce assets ahead of others, which becomes an advantage if the asset is married to the geographic area, especially if it is difficult to remove. Furthermore, first-mover locations can attract firms, organizations, scientists, and researchers related to their domain [5]. Thus, in addition to the expertise being developed within the jurisdiction, others with relevant expertise and interest may move to the location to exploit the scarce assets supported by first-mover policy.

First-mover STI policy fosters the early diffusion of knowledge and technology throughout the network of actors in the national (or regional) innovation system. Specifically, governments that enact first-mover STI policy encourage localized knowledge spillovers among the co-located firms and actors and thus, accelerate the growth of the local web of knowledge. An early concentration of spillovers can grow exponentially leading to regional disparities over time [51–53]. Moreover, institutional theory suggests that first-mover STI policies may establish a reputation associated with the early-stage technology [54], which helps build legitimacy for the area and the new technology and further expands the resource base [55, 56]. Finally, similar to other investments, these types of scarce assets (i.e., clustered knowledge of a nascent domain) do not relocate easily, but rather remain in the same place and continue to entice others to the area, enriching its innovation system. Whether promoting the creation of scarce assets (e.g., infrastructure or a dense knowledge network) or attracting additional scarce assets to the area, governments that enact early STI policies potentially build an advantage by setting these processes in motion before others do.

First-mover firms benefit from heightened buyer switching costs when customers who have adopted their products or services consider the offerings of later movers prohibitively expensive in terms of time, capital, learning, and the like, making the first-mover more attractive [44, 57]. This may seem an odd concept to apply to countries, but it is relevant. When a country builds a strong infrastructure supporting a nascent domain, they may inhibit companies and organizations reliant on this infrastructure from moving to other locations. This is also true for human capital that might be reticent to relocate [35].

#### First-mover disadvantages

While first-mover firms may enjoy certain advantages, they are also susceptible to four types of disadvantages: free-ridership, technological uncertainty, enabling new entrants, and incumbent inertia. Free-ridership is the ability of later entrants to gain from the R&D, buyer education, and infrastructure development already created by the first-movers. This activity reduces the amount of resources that later-stage entrants must dedicate to building the market. In terms of countries, first-movers must expend considerable resources to launch an infrastructure to support a nascent technological domain. Indeed, innovation policies are not protected IP and their imitation is well documented [58], allowing other countries to mimic first-mover countries, likely at a lesser cost. Later-mover governments and policy makers can gain by learning from the successes and failures of first-movers just by reading the news and policy briefings. Thus, countries enacting later-stage policy may be more selective in their infrastructure development and may not need the same level of investments. Furthermore, first-movers develop assets that are not location-specific such as knowledge and human capital that could spillover or simply move to other areas [52]. First-mover initiatives may support education and training for people who may not remain in that locale. Thus, to the extent that these infrastructure assets are not exclusive to a particular area, other locations may leverage them.

First-mover STI policy can enable later-stage policy development in other ways, which is both a positive and negative aspect. On the one hand, successfully building the initial market invites others to join, snowballing legitimacy and adoption. In a global economy, this type of action can open up opportunities for complementary activities across national borders. On the other hand, more actors intensify competition for resources such as highly trained scientists and researchers. Similarly, first-movers operate in emerging domains that are inherently uncertain and lack legitimacy, which constrains policy makers and the pioneering STI policies they enact. When trying to support a nascent domain, policy makers face opposition by others who do not accept or value the emerging technology. First-movers must develop institutions and routines to reduce uncertainty inherent with nascent development [59], that later countries can exploit. Their very existence in this field and their subsequent actions to build the market reduces technology uncertainty. Indeed, during this period, early policy efforts may fund research that fails to advance. These efforts are expensive and resource draining. Likewise, policy makers may enact ineffective solutions as they struggle to identify the appropriate mix of policy initiatives for a new domain (e.g., [21]), especially when the first-mover engages in a technology race between competing platforms or designs. Later entrants, especially a ‘fast-second,’ can benefit from the first-mover’s progress on the new technology by pursuing the market once uncertainty diminishes and platforms, standards, and technological trajectory resolve [60]. Although later-stage countries must establish their own infrastructure, early movers lay the foundation of domain legitimacy, making later developments easier to justify.

As market building is resource-intensive, ‘incumbent inertia’ may plague first-movers, limiting a firm’s ability to adapt [61]. Governments with first-mover STI policy can suffer from incumbent inertia and remain stuck with outdated approaches to an emerging field. While the early initiatives may be appropriate in the very early stages, they may become less applicable as the technology and related marketplace develop. Inertial pressures may prevent policy makers from realigning STI policies to meet new, emergent needs even as the understanding of the domain improves. Incumbent inertia can make it difficult to end poorly performing projects and shift to better projects. A lack of realignment can result in pioneers missing opportunities while jurisdictions with better-suited, later-stage policies seize them.

This brings us to a related disadvantage of first-mover governments in an uncertain technological environment: a lack of talented human resources experienced with the technology. After a breakthrough, very few people are familiar with the innovation and or have the skill and knowledge to support its development. Thus, depending on the novelty of the technology, in can be difficult to locate people who were familiar enough with technology emergence to effectively make decisions and implement first-mover policies. The lack of knowledge may lead to first-mover policies that are not aligned with the idiosyncratic needs of the technology’s development.

#### Research gap

Although the First Mover Advantage perspective was originally applied to the competitive advantage of market entry timing for firms, as discussed, it may be useful in improve our understanding of national STI policy timing. Overall, STI policies can support the development of a cluster of firms, organizations and actors that together produce technological leadership, scarce asset preemption, heightened switching costs, knowledge spillovers and an enhanced reputation for innovation. Early policies jump-start these processes before other jurisdictions and set in motion an evolving and potentially regenerative innovation system that promotes technological and economic growth. Policy feedback and interaction can cultivate a long-term strategy to attend to themes of national interest [62] or related areas [26]. As a result, pioneering policies to support STI may stimulate better innovation outcomes within their territories. Nevertheless, the benefits of first-mover STI policies may be short-lived. Information is not isolated to the country of origin and socio-technical change instigated by policy mix evolution can provide fodder for the development of policies in other countries. Policy-makers in other regions may free ride, enjoy the alleviation of technological uncertainty and newly imbued legitimacy and, with their own STI policies, enter the market in greater numbers. Nations and regions enacting first-mover STI policy may potentially suffer incumbent inertia, limiting their ability to nimbly create policies appropriate for a developing technology.

## Methods

### Setting: Nanotechnology

To gain insight into the role of STI policy timing in the emergence of a nascent technology, we focus on national policies that support nanotechnology science, technology, and innovation. Nanotechnology is the control and manipulation of atoms and molecules smaller than 100 nanometers that are used to create new materials and devices with novel properties and functions based on their small size [63]. Importantly, nanotechnology requires the ability to see and manipulate matter at the nanoscale, which was not achieved until the invention of the scanning tunneling microscope in 1981 [64]. Thus, 1981 acts as a robust starting date for the examination of policy impacts on entrepreneurial and innovative activity. Importantly, nanotechnology was not explicitly supported by STI policy until the 1980s, and thus, we can observe the starting point for this area of STI policies without censoring.

Nanotechnology intersects several scientific disciplines including chemistry, physics, biology, robotics, and computer science [65] and has permeated into several industries including textiles, biotech, automobiles, optics, pharmaceuticals, printing, and dentistry [66, 67]. Indeed, by the end of 2019, almost 9000 commercial products used nanotechnology throughout a spectrum of industries from cosmetics to construction materials, scientific instrumentation to sporting equipment. This setting is a particularly relevant setting for our research question since corresponding policy spans science, technology, and innovation policies.

### Data

This study focuses on policies specifically designed to support and spur the science, technology, and innovation activities of a nation. We constructed a database of national-level nanotechnology-related STI policies and related outcomes from over 20,000 pages of archival data from over 1,200 documents and websites. Sources include a wide range of organizations such as governments, associations and groups, universities, media, market research firms, and nanotechnology firms. Examples include the U.S.’s National Science Foundation; Japan’s Ministry of Education, Culture, Sports, Science and Technology; Chinese Academy of Sciences; U.K.’s Royal Society and Department for Environment, Food and Rural Affairs; European Commission; Canada’s National Institute for Nanotechnology; Organisation for Economic Co–operation and Development; WorldBank; and United Nations’ Educational, Scientific & Cultural Organization.

**Fig 1:**
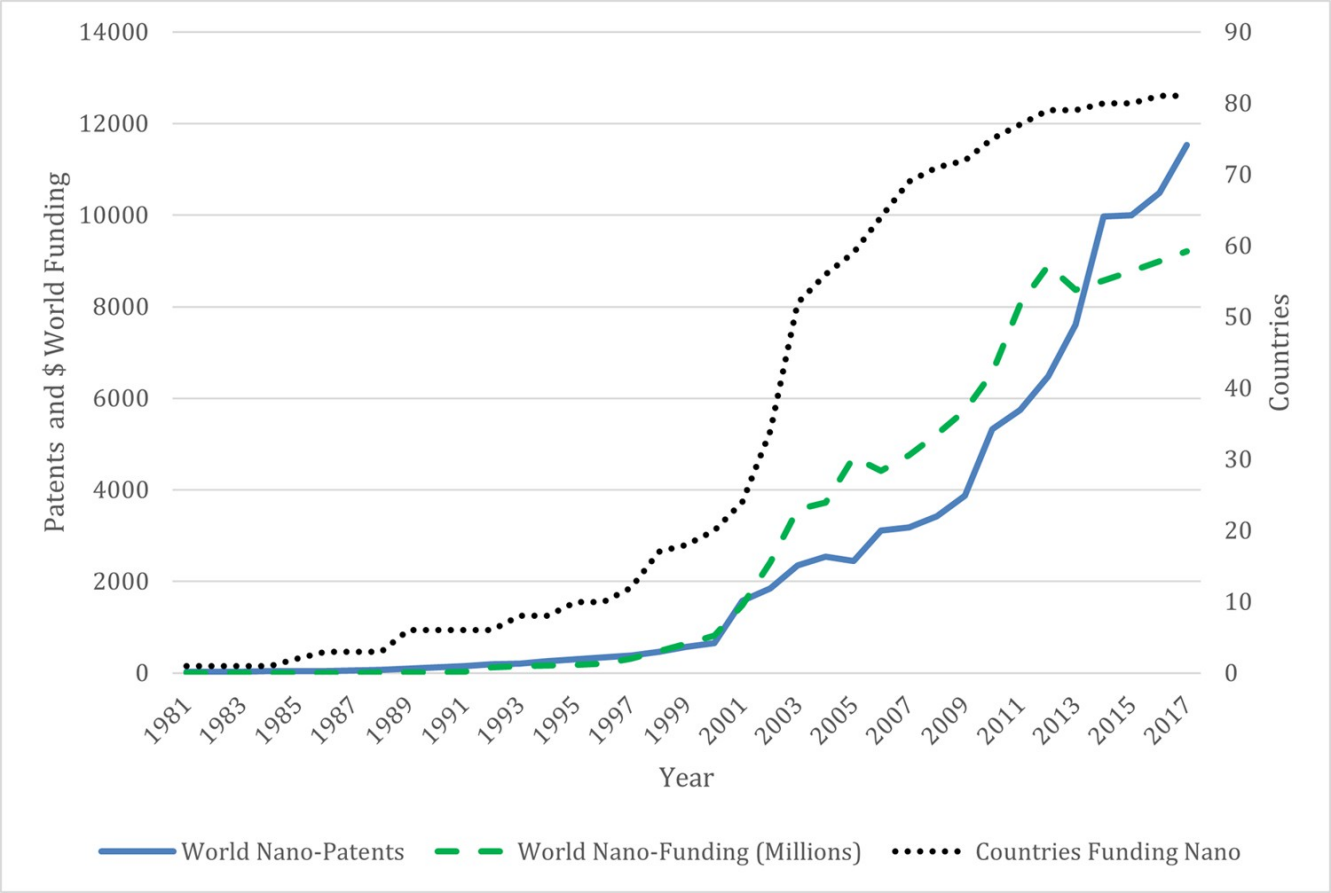
Summary of world nanotechnology patenting and government funding annually, 1981–2017.

By the end of 2017, 81 countries had national STI policy initiatives for nanotechnology, the growth of which is depicted by the dotted line in Fig 1. Table 2 provides examples of nanotechnology STI policy initiatives. The earliest national nanotechnology STI policy emerged in Japan’s ERATO Nanostructure and Ultrafine Particle programs in 1981. The U.S. Department of Energy followed with policies in 1985 as did the U.K.’s National Physical Laboratory and Department of Trade and Industry (DTI) in 1986. More recent policies have appeared in the Dominican Republic’s Strategic Plan of Science, Technology, and Innovation Initiative in 2008 and Panama’s program through the National Secretary of Science, Technology and Innovation in 2010. The number of firms in each country could not be verified for ten countries and were eliminated from analysis. The final sample of 71 countries represents over 99 percent of all nanotechnology patents worldwide. We ran several robustness checks. For example, we ran analyses including all countries and found no difference in the results. We replicated the models using countries that had no nanotechnology related firms, patents, or publications and found similar results to those detailed here. To focus on countries that participate in the field, we report the findings for the sample. Given the extensive data collection, it is unlikely that STI policies regarding nanotechnology were not included; however, it is possible that unpublicized initiatives were missed. This study follows methods used by previous work using nanotechnology data to examine policy dynamics in innovation (e.g., [68, 69]) and entrepreneurship (e.g., [5, 70]).

**Table 2:** Examples of first-mover and later-stage national nanotechnology STI policy program.

First-Movers		Later-Stage	
Japan (1981)	ERATO’s Nano-structure and Ultra-fine particles programs	Austria (2004)	Austrian Nano Initiative
U.S. (1985)	Office of Basic Energy Sciences, DOE programs	Columbia (2004)	Advanced Materials and Nanotechnology Strategic Initiative
U.K. (1986)	National Physical Laboratory and the DTI’s National Initiative on Nanotechnology	Indonesia (2005)	Masyarakat Nano Indonesia
China (1986)	MOST’s Climbing Project on Nanometer Science	Peru (2006)	National Strategic Plan for Science, Technology and Innovation for Human Competitiveness and Development
Japan (1988)	ERATO’s Quantum Wave Project	Uruguay (2006)	Uruguay Nanotechnology Group
U.K. (1988)	LINK Nanotechnology Programme	Dominican Republic (2008)	Strategic Plan of Science, Technology and Innovation Initiative
Canada (1990)	Semiconductor Nanostructure Project	Panama (2010)	National Secretary of Science, Technology and Innovation

### Dependent variables

The study of STI policy performance requires internationally comparable indicators, which can be difficult to obtain [71]. We use multiple dependent variables to measure entrepreneurial activity and innovation, cultivated from several data sources. First, we looked at entrepreneurship using the number of nanotechnology firms started in each country as reported in the Nanowerk database. The most recent year of complete data for all countries is 2017, which was used for this study. To examine the relative level of nanotechnology specific entrepreneurship activity in a country, we calculated *Nano Firms/All Firms (Country)*: the ratio of nanotechnology firms to the number of all new firms founded in a country according to the Worldbank Database [72]. This ratio provides insight into how far nanotechnology entrepreneurship has diffused through a country. A more traditional measure of technology venturing such as the ratio of nanotechnology dedicated firms to all existing firms in a country was sought; however, countries differed in their reporting and definitions of companies, which made the total number of firms not comparable. The WorldBank reliably measures the number of new firms per country each year; thus, we relied upon these data. Next, we calculated *Nano Firms Country/World*: the proportion of nanotechnology firms in the country compared to all nanotechnology firms in the world to construct a competitive measure of venturing.

To evaluate the level of innovation within each country, we examined patents and publications. Scholars often use patent data to measure innovation (e.g., [73–77]). Although imperfect [78], patents represent innovative activity because they signal novel technical accomplishment and competence [79] since they reflect a coalescence of resources, skills, and know-how. To measure comparative advantages among countries in resources, skills and knowledge within this nascent domain, patents serve as a reasonable proxy.

In 2011, all patent offices around the world implemented a standardized classification system for nanotechnology related patents. We collected the number of nanotechnology related patents granted by the European Patent Office (EPO) and the United Stated Patent and Trademark Office (USPTO) from Statnano, a reliable database of nanotechnology related statistics [80]. To use the latest available complete data and parallel the entrepreneurship measure, we collected data for the year 2017. To measure a country’s level of nanotechnology patenting relative to other countries, we calculated *Nano Patents*: *Country/World*: the count of nanotechnology patents for each country was divided by the total number of nanotechnology patents in the world granted by the EPO and USPTO. As a comparison measure, we calculated *Nano Patents per capita*: by dividing the number of nanotechnology patents for each country by its population to determine a per capita innovation value for patenting activity.

Another way to measure innovation at the country level is through publications. We collected data on academic publications in nanotechnology-related areas from the Statnano database. To compare a country’s publication activity relative to other countries, we calculated *Nano Publications*: *Country/World*: the number of nanotechnology publications for each country was divided by the number of nanotechnology publications in the world, as indexed by ISI-Web of Science. Again, to use the latest available complete data and parallel the entrepreneurship measure, we collected data for the year 2017. As a comparison measure, we calculated *Nano Publications per capita*: by dividing the number of nanotechnology publication for each country by its population to determine a per capita innovation value for publishing activity. As a robustness check we analyzed the data using the absolute numbers instead of relative proportions. Unsurprisingly, these models provided similar, but amplified results.

### Independent variables

To measure the timing of STI policies, we use the year of the country’s first implementation of national nanotechnology initiatives: *Year of First National Nanotech Program*. Second, we evaluate the amount of government nanotechnology R&D funding (one type of STI policy program) by each nation in 1990: *National Nano R&D Funding in 1990*. The first six countries to enact nanotechnology STI policies did so before 1990. There was a break in the pattern of policy adoption and no other countries passed related policies until 1993. Thus, 1990 is a natural break point for analysis and helps identify early enactors of related initiatives.

Third, to examine consequences of later-stage STI policy, we used the nation’s nanotechnology STI funding in 2003: *National Nano R&D Funding in 2003*. This measure was adopted three years after the announcement of the National Nanotechnology Initiative (NNI) in the U.S. that triggered governments around the world to start related funding efforts. The NNI announcement distinguishes the time when nanotechnology achieved widespread legitimacy and recognition. Fig 1 shows that the number of countries announcing new national nanotechnology policies greatly increased between 2002 and 2003, indicating a strong shift in government funding trends. Therefore, funding amounts in 2003 logically measure later-stage STI policy actions. Moreover, national level funding data in 2003 covered all of the countries in the sample, while data for other years were available for less than 80%. As a robustness check, we examined other years of nanotechnology funding for robustness and obtained consistent results. We also measured the total amount of national nanotechnology funding through 2003 (*Cumulative National Nano R&D Funding through 2003*) to gain insight into the influence of the cumulative amount of funding on our outcome variables. All independent variable measures were standardized except the first program year.

### Control variables

While testing the effects of timing of national STI policies on nanotechnology advancement and entrepreneurship, we parse out potential alternative factors. Since the level of R&D funding in a country influences the level of innovation, we control for the gross domestic expenditures on R&D as a percentage of the country’s gross domestic product (GDP), indicated as ‘*R&D Intensity*’ as reported by UNESCO. We controlled for the countries’ sizes using its population. Two additional variables: ease of starting a business and international IP protection were included to control for a country’s relative infrastructure for entrepreneurship and innovation. The ‘ease of starting a business’ measure is an index generated by the World Bank that calculates a country’s regulation infrastructure to support new business economic activity: starting a business, permits, employment, property registration, taxes, international trade, contract enforcement, investor protection, obtaining electricity, obtaining credit, and closing a business. The international IP protection measure is calculated by the Global Innovation Policy Center and accounts for five sets of indicators that indicate a country’s IP environment (see www.theglobalipcenter.com). Per capita models did not include the population control, as it was included in the dependent variables. All control variables were collected for 2014, the latest year of complete data for all countries and allows for a three-year lag. Finally, we standardized the control variables as recommended by Aiken and West [81].

### Analysis

Our analytic strategy involves a country level regression on a relatively small sample.

Thus, we used generalized linear models (GLM) in Stata 15 to analyze the relationship between national nanotechnology STI policy and relevant entrepreneurship and innovation [82, 83], which is an extension of the general linear models that allows for a non-normal distribution of dependent variables or residuals [84]. Each dependent variable was modeled separately. To reduce the influence of heteroscedasticity, the models estimate robust standard errors. As a robustness check, we also modeled the data using a general linear regression; however, this requires residuals to be distributed normally, which was not universally the case. Nevertheless, general linear models provided an r–squared of over 0.55 indicating strong results for these models.

## Results

Fig 1 shows the amount of government national nanotechnology STI funding annually from 1981 through 2017. The figure shows a distinct pattern in policy adoptions, indicating a sharp increase around the world after 2000. Those nations that established initiatives in the 1980s acted at least a full decade before the bandwagon of national initiatives created after the announcement of the NNI in 2000. Thus, countries that enacted initiatives 1980s and 1990s are early-movers in nanotechnology policy.

Table 3 shows the correlation matrix and descriptive statistics of the non-standardized data. Six sets of models provide a more nuanced understanding of the temporal dynamics discussed above. Table 4 reports the relationship of national nanotechnology STI policy timing and the relative level of nanotechnology entrepreneurship activity in a country by the proportion of a country’s new firms that were focused on nanotechnology. Model 1 includes only the control variables and indicates that higher levels of national R&D intensity and IP protection are related to higher levels of firms. However, ease of starting a business in a country was negatively related to corresponding entrepreneurship. Model 2 adds the year of a country’s first nanotechnology policy and shows that the year of funding did not influence the related entrepreneurship relative to other firms in the country. Model 3 provides more insight by including a country’s amount of national funding on nanotechnology in 1990 and 2003 and shows that countries with a high level of later funding had more nanotechnology dedicated firms relative to other firms in the country. Model 4 shows that countries with a higher cumulative funding also had a higher proportion of nanotechnology ventures in the country. These models suggest that countries can benefit by later funding programs.

**Table 3:** Correlation matrix and descriptive statistics.

	Variable	Mean	Std. Dev.		Min	Max	1	2	3	4	5
1	Nano Firms/All Firms (Country)	0.39	0.80		0	4.03	1.00				
2	Nano Firms Country/World	0.01	0.06		0	0.47	0.35	1.00			
3	Nano Patents: Country/World	1.40	5.48		0	43.79	0.33	0.94	1.00		
4	Nano Patents per capita	3.54	7.29		0	43.92	0.25	0.25	0.35	1.00	
5	Nano Publications: Country/World	1.74	4.76		0	36.33	0.15	0.44	0.48	0.11	1.00
6	Nano Publications per capita	65.62	73.54		0	426.57	0.24	0.08	0.11	0.59	0.05
7	R&D Intensity	1.25	1.10		0	4.29	0.54	0.27	0.36	0.57	0.28
8	Population	81.96	226.13		0.55	1399.45	0.04	0.19	0.19	-0.09	0.80
9	Ease of Starting Business	85.19	8.80		59.13	99.96	-0.07	0.07	0.11	0.30	-0.16
10	International IP protection	5.92	1.51		2	8.50	0.42	0.25	0.22	0.54	0.12
11	VC Funding/GDP	0.34	0.72		0	3.80	0.21	0.60	0.58	0.16	0.47
12	Year of First National Nanotech Program	2001.75	6.74		1981	2018	-0.46	-0.44	-0.47	-0.33	-0.52
13	National Nano R&D Funding in 1990	0.30	1.40		0	10.40	0.50	0.24	0.32	0.09	0.30
14	National Nano R&D Funding in 2003	50.29	147.19		0	862	0.57	0.74	0.83	0.31	0.54
15	Cumulative National Nano R&D Funding through 2003	148.30	531.44		0	3167.6	0.60	0.73	0.81	0.28	0.40
	Variable	6	7	8	9	10	11	12	13	14	15
6	Nano Publications per capita	1.00									
7	R&D Intensity	0.72	1.00								
8	Population	-0.16	0.01	1.00							
9	Ease of Starting Business	0.47	0.35	-0.48	1.00						
10	International IP protection	0.70	0.71	-0.09	0.44	1.00					
11	VC Funding/GDP	0.10	0.43	0.45	0.00	0.25	1.00				
12	Year of First National Nanotech Program	-0.45	-0.61	-0.26	-0.19	-0.62	-0.39	1.00			
13	National Nano R&D Funding in 1990	0.01	0.27	0.16	0.03	0.23	0.13	-0.58	1.00		
14	National Nano R&D Funding in 2003	0.15	0.46	0.24	0.10	0.33	0.45	-0.64	0.72	1.00	
15	Cumulative National Nano R&D Funding through 2003	0.10	0.41	0.15	0.10	0.30	0.41	-0.59	0.75	0.98	1.00

**Table 4:** Generalized linear models for nanotechnology firms in a country compared to all firms in the country.

Variables	Model 1		Model 2		Model 3		Model 4	
Year of First National Nanotech Program			-0.031					
			(0.024)					
National Nano R&D Funding in 1990					0.103			
					(0.104)			
National Nano R&D Funding in 2003					0.229	*		
					(0.094)			
Cumulative National Nano R&D Funding through 2003							0.331	***
							(0.056)	
R&D Intensity	0.418	*	0.365	**	0.307	**	0.328	**
	(0.165)		(0.147)		(0.121)		(0.124)	
Population	-0.100		-0.151	*	-0.159	**	-0.108	*
	(0.063)		(0.072)		(0.06)		(0.052)	
Ease of Starting Business	-0.735	*	-0.759	*	-0.769	*	-0.736	*
	(0.313)		(0.318)		(0.324)		(0.315)	
International IP protection	0.214	^	0.099		0.186		0.180	
	(0.13)		(0.158)		(0.118)		(0.119)	
VC Funding / GDP	-0.013		-0.019		-0.059		-0.113	^
	(0.109)		(0.092)		(0.064)		(0.064)	
Constant	0.407	***	62.261		0.355	**	0.350	**
	(0.12)		(48.967)		(0.123)		(0.122)	
Log pseudolikelihood	-66.068		-64.142		-53.921		-51.532	
Residual df	65		64		63		64	

Notes: n = 71, Significance levels: ^ 0.10, *0.05, **0.01, and ***0.001.

Robust standard errors reported in parentheses.

Table 5 reports the relationship between national nanotechnology STI policy timing and proportion of nanotechnology firms in the country compared to all nanotechnology firms in the world. Model 6 shows that countries that started their programs later had a smaller proportion of nanotechnology firms relative to the world. However, Model 7 indicates that higher funding early on led to a lower proportion of the world’s nanotechnology entrepreneurship while later funding increases the amount of relative nanotechnology entrepreneurship. Model 8 shows that countries with a higher cumulative amount of funding through 2003 had a higher proportion of nanotechnology firms, but the influence was marginally significant.

**Table 5:** Generalized linear models for proportion of country’s nanotechnology firms to world total.

Variables	Model 5		Model 6		Model 7		Model 8	
Year of First National Nanotech Program			-0.003	*				
			(0.002)					
National Nano R&D Funding in 1990					-0.024	*		
					(0.011)			
National Nano R&D Funding in 2003					0.052	***		
					(0.011)			
Cumulative National Nano R&D Funding through 2003							0.029	^
							(0.000)	
R&D Intensity	-0.007		-0.013		-0.019	***	-0.015	^
	(0.01)		(0.011)		(0.006)		(0.008)	
Population	-0.004		-0.010		-0.008	*	-0.005	
	(0.01)		(0.01)		(0.003)		(0.006)	
Ease of Starting Business	-0.001		-0.003		-0.008		-0.001	
	(0.008)		(0.008)		(0.006)		(0.007)	
International IP protection	0.013		0.001		0.015	*	0.010	^
	(0.011)		(0.008)		(0.006)		(0.006)	
VC Funding / GDP	0.032		0.032		0.017	*	0.023	^
	(0.025)		(0.023)		(0.007)		(0.014)	
Constant	0.009	^	6.574	*	0.002		0.004	
	(0.005)		(3.157)		(0.003)		(0.004)	
Log pseudolikelihood	119.242		123.381		163.16		142.166	
Residual df	65		64		63		64	

Notes: n = 71, Significance levels: ^ 0.10, *0.05, **0.01, and ***0.001.

Robust standard errors reported in parentheses.

Table 6 reports the influence of national nanotechnology STI policy timing on nanotechnology-related patenting in a country per capita. Model 10 showed no influence of timing; however, Model 11 shows that higher levels of funding early on lead to lower patenting per capita, while funding later (2003) led to higher patenting. Table 7 reports the models for a country’s share of the world’s nanotechnology patents. Model 14 shows that the later a country enacted an initiative, the lower the proportions of related patents. However, similar to Table 6, Model 15 indicates that higher amounts of funding early on lower the proportion of patents while higher amounts later funding increases it. Model 16 shows that the cumulative funding positively influences the amount of patenting. This may suggest benefits of funding STI policies later in a technology’s development.

**Table 6:** Generalized linear models for a country’s nanotechnology patents per capita.

Variables	Model 9		Model 10		Model 11		Model 12	
Year of First National Nanotech Program			0.115					
			(0.146)					
National Nano R&D Funding in 1990					-1.827	***		
					(0.483)			
National Nano R&D Funding in 2003					2.151	***		
					(0.572)			
Cumulative National Nano R&D Funding through 2003							0.530	
							(0.762)	
R&D Intensity	2.830	^	3.019	*	2.561	^	2.685	^
	(1.554)		(1.506)		(1.395)		(1.56)	
Ease of Starting Business	0.704		0.551		0.334		0.719	
	(0.912)		(1.021)		(0.9)		(0.89)	
International IP protection	2.284		2.728		2.485		2.227	
	(2.459)		(2.838)		(2.38)		(2.467)	
VC Funding / GDP	-0.502		-0.388		-1.214	**	-0.669	
	(0.818)		(0.847)		(0.482)		(0.655)	
Constant	1.705	***	-228.748		1.518	**	1.608	***
	(0.463)		(292.958)		(0.514)		(0.496)	
Log pseudolikelihood	-225.002		-224.672		-222.16		-224.651	
Residual df	66		65		64		65	

Notes: n = 71, Significance levels: ^ 0.10, *0.05, **0.01, and ***0.001.

Robust standard errors reported in parentheses.

**Table 7:** Generalized linear models for a country’s share of world nanotechnology patents granted.

Variables	Model 13		Model 14		Model 15		Model 16	
Year of First National Nanotech Program			-0.328	*				
			(0.154)					
National Nano R&D Funding in 1990					-2.180	**		
					(0.762)			
National Nano R&D Funding in 2003					5.241	***		
					(0.925)			
Cumulative National Nano R&D Funding through 2003							3.099	*
							(1.988)	
R&D Intensity	0.662		0.093		-0.626		-0.190	
	(1.146)		(1.215)		(0.603)		(0.965)	
Population	-0.090		-0.628		-0.472		-0.167	
	(0.965)		(0.947)		(0.299)		(0.492)	
Ease of Starting Business	0.888		0.640		0.113		0.879	^
	(0.639)		(0.614)		(0.478)		(0.495)	
International IP protection	-0.330		-1.550		-0.220		-0.654	
	(1.198)		(0.984)		(0.592)		(0.829)	
VC Funding / GDP	2.567		2.500		1.023		1.632	
	(2.434)		(2.207)		(0.681)		(1.226)	
Constant	0.675		657.507	*	-0.054		0.139	
	(0.483)		(309.334)		(0.229)		(0.302)	
Log pseudolikelihood	-205.091		-200.608		-147.805		-173.595	
Residual df	65		64		63		64	

Notes: n = 71, Significance levels: ^ 0.10, *0.05, **0.01, and ***0.001.

Robust standard errors reported in parentheses.

Table 8 turns to the influence of national nanotechnology policy timing on related publications per capita. Model 18 shows no influence of the year of the first national nanotechnology STI program. However, Model 19 shows that higher levels of funding early on lead to lower publishing per capita, with marginal significance. Model 20 indicates that a higher cumulative amount of funding led to significantly lower publications per capita. Lastly, Table 9 reports the models for a country’s share of the world’s nanotechnology publications. Model 22 suggests a first-mover advantage for those countries that funded nanotechnology innovation early on: the later the first initiative, the smaller the share of nanotechnology publications. This supports the proposition of advantages for countries with early STI policies in the technology. However, Model 23 shows that higher funding in 2003 was related to a higher share of publications suggesting that the amount of funding matters more than the timing of it. Countries with higher overall funding had higher shares of nanotechnology publications, but the effect was marginal (Model 24).

**Table 8:** Generalized linear models for a country’s nanotechnology publications per capita.

Variables	Model 17		Model 18		Model 19		Model 20	
Year of First National Nanotech Program			0.344					
			(1.153)					
National Nano R&D Funding in 1990					-12.082	^		
					(6.624)			
National Nano R&D Funding in 2003					0.369			
					(7.78)			
Cumulative National Nano R&D Funding through 2003							-10.852	*
							(5.084)	
R&D Intensity	38.280	**	38.845	***	41.398	***	41.247	**
	(6.752)		(6.016)		(5.513)		(5.755)	
Ease of Starting Business	23.141	*	22.686	*	19.924	*	22.833	**
	(9.728)		(9.798)		(8.846)		(9.088)	
International IP protection	27.605	**	28.933	**	29.786	**	28.763	**
	(10.696)		(10.887)		(11.272)		(11.068)	
VC Funding / GDP	-13.484	**	-13.141	**	-13.637	*	-10.079	^
	(4.458)		(5.174)		(6.293)		(5.77)	
Constant	38.634	***	-650.309		40.887	***	40.630	***
	(3.695)		(2310.31)		(3.678)		(3.466)	
Log pseudolikelihood	-367.624		-367.572		-363.594		-364.891	
Residual df	66		65		64		65	

Notes: n = 71, Significance levels: ^ 0.10, *0.05, **0.01, and ***0.001.

Robust standard errors reported in parentheses.

**Table 9:** Generalized linear models for a country’s share of world nanotechnology publications granted.

Variables	Model 21		Model 22		Model 23		Model 24	
Year of First National Nanotech Program			-0.217	**				
			(0.083)					
National Nano R&D Funding in 1990					-0.696			
					(0.432)			
National Nano R&D Funding in 2003					1.781	***		
					(0.439)			
Cumulative National Nano R&D Funding through 2003							0.888	^
							(0.543)	
R&D Intensity	1.138	*	0.761	*	0.688	*	0.894	*
	(0.476)		(0.38)		(0.303)		(0.416)	
Population	3.497	**	3.141	**	3.358	**	3.475	**
	(1.161)		(1.082)		(1.074)		(1.144)	
Ease of Starting Business	2.186	**	2.021	**	1.922	**	2.183	**
	(0.836)		(0.757)		(0.784)		(0.841)	
International IP protection	-0.478		-1.286	*	-0.448		-0.571	
	(0.507)		(0.66)		(0.414)		(0.457)	
VC Funding / GDP	-0.126		-0.170		-0.645		-0.394	
	(0.746)		(0.619)		(0.454)		(0.531)	
Constant	0.407		435.266	**	0.155		0.253	
	(0.287)		(166.508)		(0.29)		(0.283)	
Log pseudolikelihood	-163.47		-156.943		-150.429		-157.445	
Residual df	65		64		63		64	

Notes: n = 71, Significance levels: ^ 0.10, *0.05, **0.01, and ***0.001.

Robust standard errors reported in parentheses.

The GLM models of the influence of the timing and amounts of national nanotechnology STI funding programs in 71 countries are summarized in Table 10. Overall, the findings are fairly consistent. Countries with national STI programs early in the development of the technology was related to lower overall patents and publications per capita and relative to the rest of the world while larger national STI programs once the technology has obtained legitimacy was related to higher entrepreneurship and innovation as measured by firms, patents, and publications. The cumulative amount of national nanotechnology funding led to higher amounts of entrepreneurial and innovation activities relative to the rest of the world, but not publications per capita.

**Table 10:** Summary of findings.

				Firms
	Firms			Patents
	*per all firms*	Patents	Publications	Publications
	*in country*	*per capita*	*per capita*	*relative to world*
Year of First National Nanotech Program				-
Early Funding—National Nano R&D Funding in 1990		-	-	-
Later Funding—National Nano R&D Funding in 2003	+	+		+
Cumulative National Nano R&D Funding through 2003	+		-	+

## Discussion and conclusions

STI initiatives are an important component of the public policy mix [18, 19, 21], in both the amount of funding and the scope of their influence. In this study, we have set out to instigate a discussion by asking if the timing of adoption of a country’s STI policies during the emergence of a nascent technology is associated with related entrepreneurship and innovation? To address this question, we crossed crossing disciplines and applied the first-mover firm perspective [44] to the domain of STI policy, a unique contribution to both fields. Like first-mover firms, first-mover countries are pioneering nations that are motivated to implement policies ahead of others to stimulate progress in a nascent science, technology, and innovation domain. Being able to enact a first-mover STI policy signals to others that the country is willing and able to take chances on supporting an emerging domain of activity. Both first-mover firms and countries design policies for entry into the nascent domain given new circumstances, risks, historical legacies, and existing infrastructure. Decision makers at firms and governments both must weigh the risk of not moving fast enough and being left behind due to lack of investment. We argue that potential advantages of enacting first-mover STI policy include technology leadership, scarce asset preemption and heightened switching costs, as well as long-term gains from reinvesting early returns, directing policy and standards, and building reputation and legitimacy. Together, these benefits may stimulate a strong national system of innovation and entrepreneurship. However, areas with a first-mover STI policy may also face disadvantages that can drain resources and, unless properly managed, limit the long-term benefits of adopting STI policies earlier than other places do.

We empirically examine the relationship between the timing of STI policy and entrepreneurship and innovation by drawing on the global emergence of nanotechnology. These analyses confirm a relationship between the timing of STI policy and a country’s innovations activities, which are summarized in Table 10. The findings reveal that countries that started funding STI programs in nanotechnology *later* had a lower proportion of the total nanotechnology firms, patents, and publications in the world. This suggests some first-mover advantages to STI policy. For example, some countries with first-mover nanotechnology policies led to the construction of advanced nanotechnology facilities for both academic and commercial work such as the Minitec research facility in France and the National Nanotechnology Users Network in the U.S. These scarce assets remain available for proximate organizations and researchers. First-mover STI policy may also enable governments to construct a long-term strategy to reduce costs associated with the technology’s development in light of an existing program such as the U.K.’s DTI created an initiative in 1986 to coordinate nanotechnology research across the country that enabled a reduction in redundancies across labs and the identification of gaps in the innovation system.

However, when the amount of funding was considered, countries with *larger early* programs had a lower proportion of the total nanotechnology firms, patents, and publication and fewer nanotechnology-related patents and publication per capita. Countries with *larger later* programs had the opposite situation such that there was a higher proportion of the total nanotechnology firms, patents, and publication in the world and more nanotechnology-related patents per capita and firms relative to other firms in the country. These findings suggest that large amounts of funding invested early in the technology’s development did not cause the sponsoring countries to reap first-mover advantages. Countries that started later or were able to build considerable support for STI policy through cumulative funding gained with higher levels of innovation and entrepreneurship. In spite of some first-mover advantages for STI policy, later-movers can gain an advantage.

At first glance it would appear that countries do not benefit from starting and funding large STI initiatives early in a technology’s development. However, early movers remain strong with above average venturing, patenting, and publishing activity. Digging deeper into the individual countries data shows that those that were neither early (before 1993), nor late (after 2003), had exceptional increases in their nanotechnology activities. For example, Singapore started a nanotechnology program in 1995 that has grown slowly over time. The country now has the highest number of nanotechnology publications per capita in the world, almost twice that of other highly publishing countries. Similarly, South Korea also started a nanotechnology program in 1995 and has the highest nanotechnology patenting per capita and a very strong share of the world’s nanotechnology patents in 2017 (12%). This indicates that second or later movers are not destined to remain behind earlier movers.

### Limitations

Measuring the efficacy of individual policy instruments is fraught with challenges including context, interactions, long lag times [12, 33, 85, 86] and additionality [25–27], to name a few. This study does not address the efficacy of a single policy, but rather considers a national STI policy initiative in a nascent domain to be a signal of support for economic and innovative activity therein. Thus, this study highlights the potential benefits and disadvantages of implementing STI initiatives at different stages across the technology lifecycle [87]. Effective implementation requires that policies match the needs of a country and the stage-specific needs of the technology’s development. For example, initiatives enacted while the technology is emerging must center on basic infrastructure development instead of commercialization activities [12, 88, 89]. Indeed, if planned strategically, first-mover and later-stage STI policy can be integrated to foster entrepreneurship and a robust, cohesive system of innovation. While this may seem intuitive, this progression has not always been the case. Thus, policy makers and researchers should consider not only if an initiative should be enacted, but also, whether it effectively matches the phase of the technology’s development and the infrastructure available. As policy and the components of the innovation system in which is it embedded are fatefully intertwined, conclusions regarding causality are plagued with inherent endogeneity [11, 90]. As with most studies, we could not control for all sources of endogeneity; however, by including a wide range of controls, we attempt to account for some of the key factors that influence entrepreneurial and innovation activities in a country.

Other limitations stem from the exploratory nature of the study and the available data. First, due to data availability, our timing measures–early vs. late–do not consider the incremental progression of policy or technological development. Future research that uses more fine-grained measures of timing could clarify the temporal dynamics seen here. Second, we cannot claim a causal relationship between policy timing and firm founding, patenting, or publication activity. As nanotechnology has only been developed in the last 40 years, certainly additional advancement will be made both in innovation and policy. It may be that the temporal dynamics shift and leaders today may be laggards in the future. Although limiting, our data do provide insight into how timing influences some of the country level outcomes. Likewise, we measure innovation at the national level with patents and publications, a measure widely used among researchers who study innovation [79]. Nevertheless, the system of discovery, development and implementation of new technologies combines complex phenomena and measuring them by patent or publications cannot capture all innovation activity. An opportunity for future research is examining the relationship between policy timing and richer measures of innovation system development such as research or new product launches.

### Implications and directions for future work

The institutions and norms of STI policies may affect not only innovation outcomes, but also *who* does the innovating [91]. As a global economy, the influence of early movers on other countries is not simply one of enabling others, but building a larger system. For example, the legitimacy and infrastructure built by the U.S.’s NNI enabled other countries to create similar later-stage initiatives deemed too risky before. Later-stage policies were implemented in countries such as India and Colombia, which explicitly used the NNI as a template for their nanotechnology STI policies. As such, countries with later-stage STI policies were bolstered by earlier countries. And although there may be reuse or recurrence of certain innovation policies across multiple countries [92, 93], local context and isomorphic differences [89] are becoming more appreciated.

Similarly, replicating best practices from one country does not result in progress in another. Innovation is a ‘cumulative process that is path-dependent and context-driven’ ([13] p. 617), with significant complementarity across various policy levers [94, 95]. As such, policy makers must take into consideration the relevant social, political, and economic contexts. Indeed, determining the right mix and timing for a country is “elusive precisely because it depends on the dynamic institutional context in which the policies are embedded” ([14] p. 1). Policy instruments used in Japan, for instance, may not be appropriate for more heterogeneous, geographically dispersed countries such as India or smaller, resource-scarce countries such as Panama. Examining policy at different levels and across nations may provide insight into establishing new firms in new domains, and the long-term development of global innovation systems. Those deciding STI policy timing must take into consideration the readiness of the context in which they will be implemented. Innovation is not a zero-sum game. Although countries are competitive, more efficient and effective policies benefit much more than the jurisdiction in which they are implemented.

In a related vein, learning and knowledge creation both within a county and among countries underpins the development of a technology globally. Now more than ever, entrepreneurship and innovation diffuse throughout the world with little regard for national boundaries [96]. As such, the STI policies that cultivate entrepreneurship and innovation in one country influence that activity in others [36]. The implementation of and results from first-mover policies form the context for later policies within and across regions. We hope that this study attracts attention to developing our understanding of how national STI policies influence entrepreneurship and innovation around the globe.
